# Alterations in pH of Coffee Bean Extract and Properties of Chlorogenic Acid Based on the Roasting Degree

**DOI:** 10.3390/foods13111757

**Published:** 2024-06-03

**Authors:** Yi Kyeoung Kim, Jae-Min Lim, Young Jae Kim, Wook Kim

**Affiliations:** 1 Department of Plant Biotechnology, Korea University, Seoul 02841, Republic of Korea; lkkim1105@korea.ac.kr; 2 Department of Chemistry, College of Natural Sciences, Changwon National University, Changwon 51140, Republic of Korea; 3 Department of Bio Health Science, College of Natural Sciences, Changwon National University, Changwon 51140, Republic of Korea

**Keywords:** coffee acidity, chlorogenic acids, UV–Vis spectrophotometer, high-performance liquid chromatography, liquid chromatography-mass spectrometry

## Abstract

Factors influencing the sour taste of coffee and the properties of chlorogenic acid are not yet fully understood. This study aimed to evaluate the impact of roasting degree on pH-associated changes in coffee bean extract and the thermal stability of chlorogenic acid. Coffee bean extract pH decreased up to a chromaticity value of 75 but increased with higher chromaticity values. Ultraviolet–visible spectrophotometry and structural analysis attributed this effect to chlorogenic and caffeic acids. Moreover, liquid chromatography-mass spectrometry analysis identified four chlorogenic acid types in green coffee bean extract. Chlorogenic acid isomers were eluted broadly on HPLC, and a chlorogenic acid fraction graph with two peaks, fractions 5 and 9, was obtained. Among the various fractions, the isomer in fraction 5 had significantly lower thermal stability, indicating that thermal stability differs between chlorogenic acid isomers.

## 1. Introduction

Coffee is the most popular and economically important beverage globally due to its unique aroma, good taste, and numerous health benefits [1]. For this reason, research on the health effects of coffee is very active [2,3,4,5,6,7]. Among the various ingredients of coffee, chlorogenic acid (CGA) can be cited as an important ingredient beneficial to health. CGA-containing foods, such as coffee, are functional foods with antioxidant properties. Six to ten percent of the dry weight of green coffee beans is made up of CGA. CGA is present at high concentrations of 70–350 mg per cup of coffee [8].

On the other hand, the flavor of coffee is determined by chemical components derived from coffee beans and the by-products generated during the roasting process [9]. Among these, compounds such as CGA, caffeine, and trigonelline contribute to the sour, bitter, and astringent taste of coffee [10,11,12]. Although extensive research has been performed on coffee taste, the mechanism of coffee taste is not yet clear.

CGA is decomposed during the roasting process; hence, its content decreases with increasing roasting degree for coffee beans [13,14]. However, studies on the effect of chlorogenic acid decomposition products on coffee taste are still at a standstill. CGA belongs to a family of related polyphenolic esters containing hydroxycinnamic acids such as ferulic acid, caffeic acid, and *p*-coumaric acid with (-)quinic acid [15,16]. Characteristically, CGA is a family of esters formed between certain *trans*-cinnamic acids and (-)-quinic acid, classified according to the number of cinnamic substituents and their esterification position in the cyclohexane ring of quinic acid. The major groups of CGA contained in green coffee beans are caffeoylquinic acid, dicaffeoylquinic acid, feruloylquinic acid, *p*-coumaroylquinic acid, and caffeoylferuloylquinic acid. Minor groups, comprising less than 1% of the total CGA content, include diferuloylquinic acid, di-*p*-coumaroylquinic acid, and dimethoxycinnamoylquinic acid [17,18].

Among the various methods developed for evaluating CGA and its derivatives in coffee beans and other plants, high-performance liquid chromatography (HPLC) [19,20,21,22], capillary electrophoresis [23,24], and micellar electrokinetic chromatography [25] are the most widely used methods. Although these methods are highly efficient for quantifying CGA and its derivatives, most of them are very expensive and involve some problems. In contrast, ultraviolet–visible (UV–Vis) spectrophotometry is a simple, fast, and inexpensive method for measuring the CGA content of coffee bean extracts. However, this method is not specific to CGA in coffee bean extracts since the CGA spectrum overlaps with caffeine [26].

Research on coffee beans has mainly focused on factors affecting taste and flavor, as well as the separation and structural characterization of compounds contained in green coffee beans, including CGA. However, the factors affecting the sour taste of coffee are still unknown. In addition, the physical properties of CGA are still unclear. Hence, this study aimed to investigate changes in the pH of coffee bean extracts depending on the roasting degree and analyze the thermal stability of CGA isomers.

## 2. Materials and Methods

**Figure 1:**
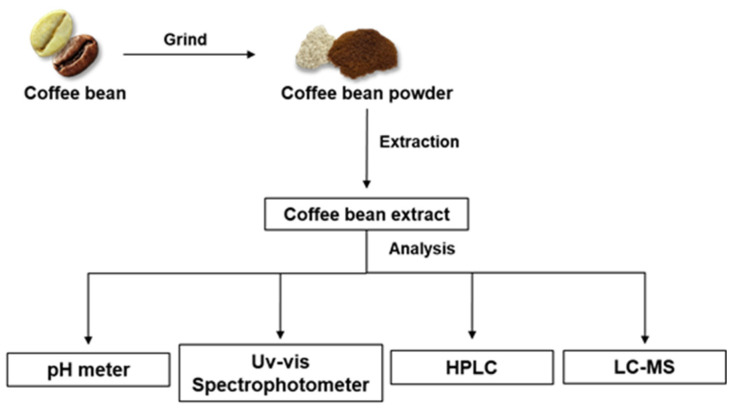
Schematic diagram showing the workflow of this study.

A schematic diagram was created to easily illustrate the workflow of this study through materials and methods (Figure 1).

### 2.1. Chemicals and Reagents

CGAs (3-*O*-caffeoylquinic acid, 4-*O*-caffeoylquinic acid, and 5-*O*-caffeoylquinic acid), caffeine, and trigonelline were purchased from Sigma-Aldrich, USA. Water and acetonitrile for HPLC were purchased from Honeywell B&J (Muskegon, MI, USA). Water and methanol for liquid chromatography-mass spectrometry (LC-MS) were also purchased from Honeywell B&J (Muskegon, MI, USA). Green coffee beans (Cameroon Blue Mountain) were collected from Oku, Cameroon, Africa.

### 2.2. Sample Prepatation

Cameroon Blue Mountain was used as the experimental green coffee bean. After roasting the green coffee beans using a roasting machine, the chromaticity value was measured using the Lighttells CM-100 Coffee Roast Degree Analyzer (Lighttells Corp. Ltd., Zhubei City, Taiwan) as the chromometer. The coffee roast index is standardized by SCAA (Specialty Coffee Association of America) and represented by Agtron number. The Agtron number corresponds to the chromaticity value. The Lighttells CM-100 Coffee Roast Degree Analyzer can measure chromaticity values from 100 to 0. If the chromaticity value is expressed as a roasting degree, 95 is a Light roasting degree, 85 is a Cinnamon roasting degree, 75 is a Medium roasting degree, 65 is a High roasting degree, 55 is a City roasting degree, 45 is a Full City roasting degree, 35 is a French roasting degree, and 25 is an Italian roasting degree. Green coffee beans have a harder outer skin than roasted coffee beans, so it is not possible to use a general coffee bean grinder. Therefore, to grind green coffee beans, a high-performance grinder was introduced that is different from the grinder for roasted coffee beans. Green coffee beans were ground using a Philips HR2602 grinder (Philips, Zhuhai, China), and the powder was filtered using a paper drip to use the filtrate. To utilize the roasted coffee bean extracts, coffee beans were ground with a Baratza Encore Coffee grinder (BGM Inc., Taichung, Taiwan), and the powders were used as the filtrate using Coffee filter FP102 (Kalita, Kagawa, Japan). All coffee bean extracts were extracted using hot water (90–95 °C) boiled with distilled water.

### 2.3. Analysis with pH Meter and UV–Vis Spectrophotometer

A pH meter (Mettler Toledo, Greifensee, Switzerland) was used to measure the pH of the coffee bean extracts. The pH measurement experiments of coffee bean extracts were repeated more than 10 times to draw graphs as mean values. Shimadzu UV-2450 (Shimadzu corp., Kyoto, Japan) was used to quantify CGA, caffeine, and trigonelline. In the case of chlorogenic acid, a 1 mg/mL stock solution was prepared, and the absorbance was measured using a solution diluted according to the concentration of the green coffee bean extract.

### 2.4. HPLC Analysis

The Waters Breeze QS HPLC system (Waters Corp., Milford, MA, USA) equipped with SunFire C18 (4.6 × 150 mm, 100 Å, 3.5 µm) column and UV–Vis detector was used for isolating the CGAs. The temperature was maintained at 25 °C. Sample elution was performed with water (mobile phase A) and acetonitrile (mobile phase B). The injection volume was 10 μL. After signal stabilization, a linear gradient elution of water (mobile phase A) and acetonitrile (mobile phase B) was applied at a flow rate of 1.0 mL/min for 30 min. The detector was set at 272 nm and 325 nm. After 1.5 mL fractions during HPLC elution, the presence or absence of chlorogenic acid was confirmed using a spectrophotometer.

### 2.5. LC-MS Analysis

CGA in the fractionated green coffee bean extracts was analyzed using ultra-performance LC-MS (UPLC-MS). The fractionated samples (10 μL) were injected using an autosampler, separated on a C18 column, and introduced into the mass spectrometer. UPLC-MS analysis was performed using a Q Exactive plus Orbitrap mass spectrometer (Thermo Fisher Scientific, Waltham, USA) at the Gyeongnam Bio and Anti-aging Core Facility Center for CWNU equipped with a Heated Electrospray Ionization (HESI) ion source at capillary voltage, 3.0 kV; capillary temperature, 250.0 °C; and S-lens RF level, 50.0. Profile mode FTMS spectra of the full negative ion were typically recorded at 70,000 resolution and collected at an *m*/*z* range of 100.0–1500.0 with 2 microscans and 150 ms maximum injection time. The higher-energy collisional dissociation tandem MS spectra for structural information were obtained using 20% normalized collision energy.

Chromatographic separation was performed using a U3000 (UltiMate 3000 UHPLC) system with a reversed-phase C18 column (Symmetry C18 Column, 100 Å, 3.5 µm, 2.1 mm × 50 mm, Waters). Sample elution was performed using a gradient program with water (A) and methanol (B). The following gradient elution program was used at a flow rate of 0.25 mL/min: 1% B, 0–5 min; 2% B, 5–20 min; 12% B, 20–30 min; 23% B, 30–40 min; 35% B, 40–50 min; 60% B, 50–60 min; 99% B, 60–65 min; and 1% B 65–90 min.

## 3. Results and Discussion

### 3.1. Changes in Coffee Bean Extract pH Depending on the Roasting Degree and Structural Analysis of Chlorogenic Acid

**Figure 2:**
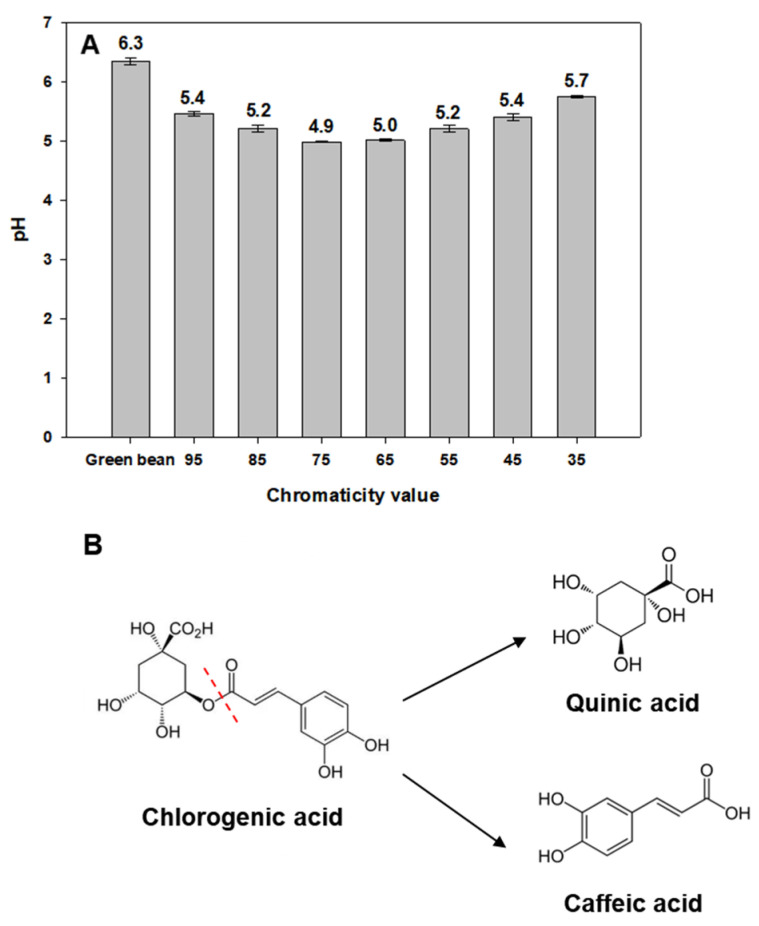
(**A**) Graphical analysis for pH change in coffee bean extract on the roasting degree. The pH of green coffee bean extract ranges from 6.3 to 6.5, depending on the water used. (**B**) Schematic diagram of the structural change of chlorogenic acid (caffeoylquinic acid) according to roasting.

In general, acidity, which indicates the degree of sourness in coffee bean extracts, decreases as the roasting degree increases. Thus, the pH of coffee bean extract increases as the roasting degree increases. To confirm this, we examined changes in coffee bean extract pH depending on the roasting degree. In contrast to conventional findings, coffee bean extract pH decreased up to a chromaticity value of 75 but increased with higher chromaticity values (Figure 2A). This phenomenon can be explained by the fact that CGA is decomposed up to a chromaticity value of 75, and the resulting decomposition products, caffeic or quinic acid, affect the pH of the coffee bean extract. Beyond a chromaticity value of 75, both CGA and its degradation products are decomposed, thus altering the pH of the coffee bean extract. Based on the structure of CGA, caffeic acid presumably has a more pronounced effect on the pH of the coffee bean extract than that of quinic acid (Figure 2B). Regardless of the cell type, the cytoplasm contains various organic acids. However, most of these organic acids are salted, and only a very small amount of free acid exists. The chlorogenic acid in green coffee beans is also salted, so it does not have a significant effect on intracellular pH (Figure 2A). Therefore, caffeic acid is produced when the ester bond of chlorogenic acid, which has an ester bond structure of caffeic acid and quinic acid, is broken down, which can affect the pH of coffee.

### 3.2. UV–Vis Absorption Spectra of Standard CGA and CGA Present in Green Coffee Bean Extract

**Figure 3:**
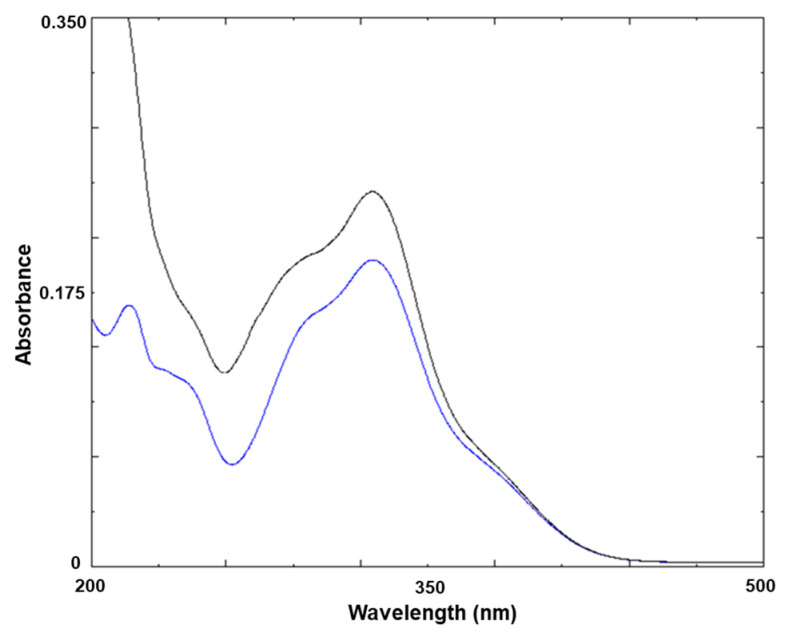
The UV–Vis absorption spectra of standard chlorogenic acid (blue) and chlorogenic acid present in green coffee bean extract (black) in the wavelength regions of 200–500 nm at room temperature. Samples were dissolved in distilled water.

A simple UV spectrum test was designed to identify CGA contained in green coffee bean extract. Figure 3 shows the UV–Vis absorption spectra between 200 and 500 nm for pure CGA and CGA present in green coffee bean extract. In this region, standard CGA showed two peak maxima. The first peak was at 217 nm with a shoulder at 238 nm, the second peak was at 325 nm with a shoulder at 296 nm, and the minimum point was at 263 nm. In contrast, the CGA spectral pattern in the green bean extract was similar to that of pure CGA, with a marginal difference in spectral band positions. This phenomenon is attributed to spectral interference between caffeine and CGA in the wavelength range of 200–500 nm [26].

### 3.3. Changes in the UV–Vis Absorption Spectrum of the Coffee Bean Extract Depending on the Roasting Degree

**Figure 4:**
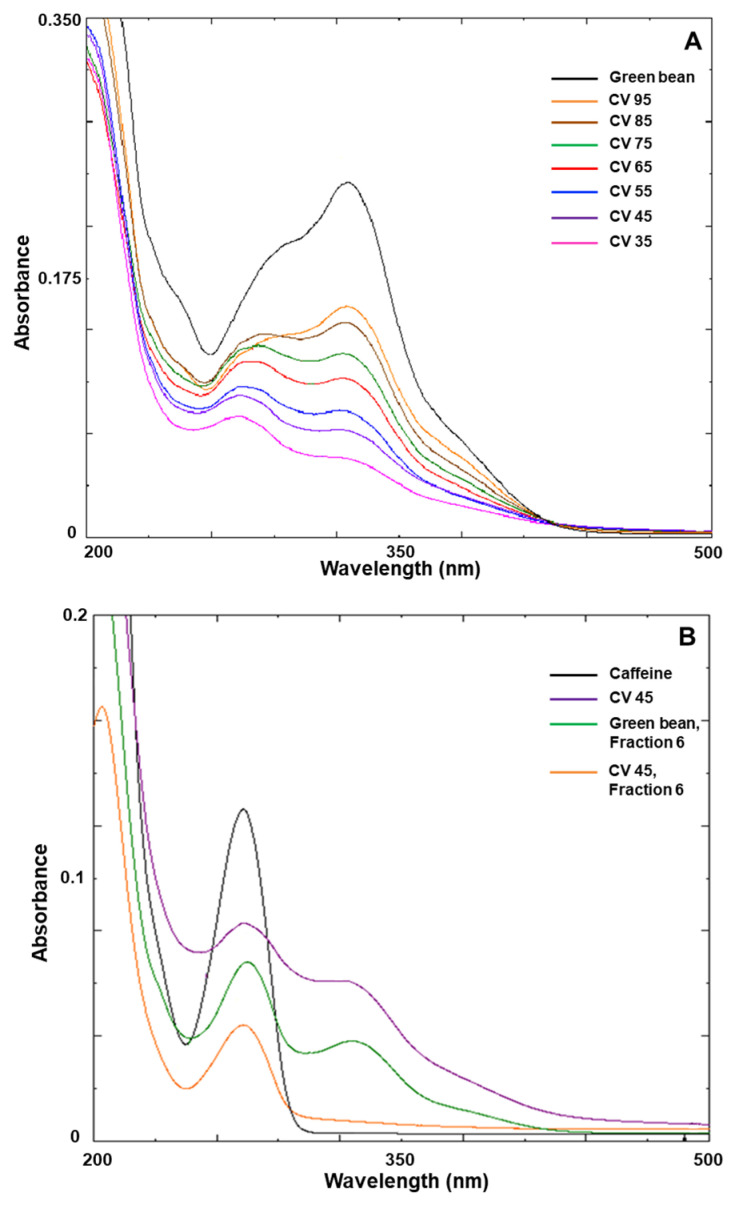
(**A**) UV–Vis absorption spectra of coffee bean extract according to roasting degree; (**B**) UV–Vis absorption spectra of caffeine and chlorogenic acid. Each spectrum represents standard caffeine (black), extract of coffee bean with a chromaticity value of 45 (purple), high-performance liquid chromatography (HPLC) fraction 6 of green bean extract (green), and HPLC fraction 6 of extract of coffee bean with a chromaticity value of 45 (orange). A 100-fold dilution of the stock solution containing 2.5 mg/mL caffeine was used for absorbance measurement. CV, chromaticity value.

Figure 4A shows changes in the UV–Vis absorption spectrum of the coffee bean extract according to the roasting degree. The spectral bands were significantly distorted with an increase in the roasting degree compared to those of the green bean extract. Two peaks were observed in the wavelength range of 200–500 nm. The first peak representing caffeine was observed between 250 and 300 nm, while the second peak representing CGA was observed between 300 and 350 nm. As shown in Figure 4A,B, as the roasting degree increased, the caffeine peak was increasingly prominent in the 250–300 nm wavelength region, while the CGA spectral band in the 300–350 nm wavelength region was significantly distorted. The peak considered as caffeine was partially purified using HPLC and coincided exactly with the peak for pure caffeine (Figure 4B). The extract obtained from coffee beans with a chromaticity value of approximately 45 was HPLC-purified and observed using a UV–Vis spectrophotometer; the caffeine peak was present, while that of CGA disappeared completely (Figure 4B). Thus, the UV–Vis spectrophotometer allows for a comparison of thermal stability between caffeine and CGA, depending on the roasting degree.

### 3.4. Separation of CGAs Using HPLC and Determination of CGA Isomers in Green Coffee Bean Extract Using LC-MS

**Figure 5:**
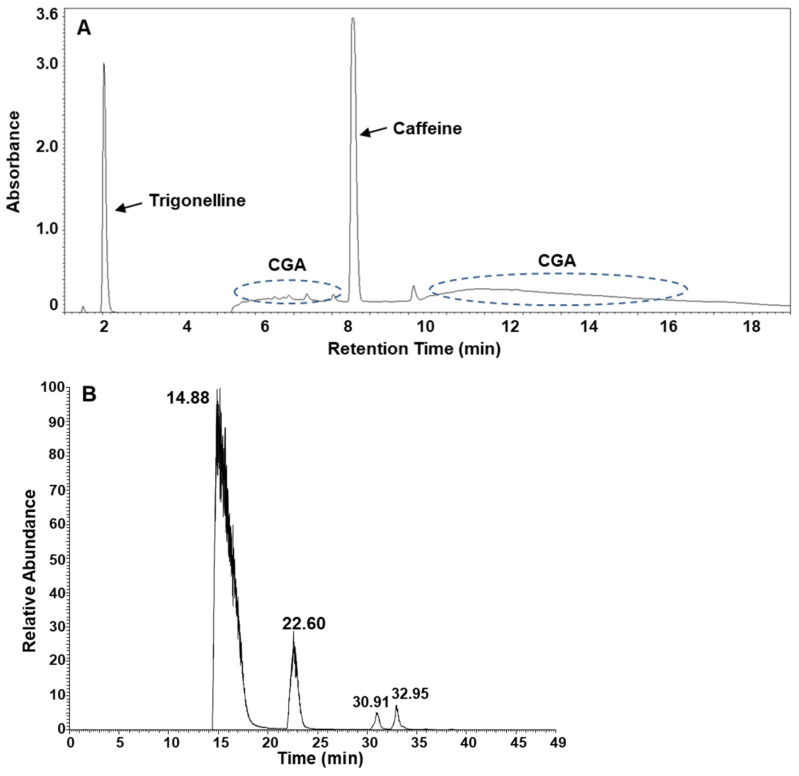
(**A**) HPLC chromatogram of green coffee bean extract; (**B**) extracted ion chromatogram of chlorogenic acid isomers in green coffee bean extract using liquid chromatography-mass spectrometry (LC-MS).

The composition of the green coffee bean extract was determined using HPLC. At least three major components, namely trigonelline, caffeine, and chlorogenic acid, were identified in the experimental system we used, as shown in Figure 5A. LC-MS was introduced to identify CGA isomers in the green coffee bean extract. Four CGA species were identified in the green coffee bean extract of Cameroon Blue Mountains (Figure 5B).

### 3.5. Analysis of Thermal Stability among CGA Isomers

**Figure 6:**
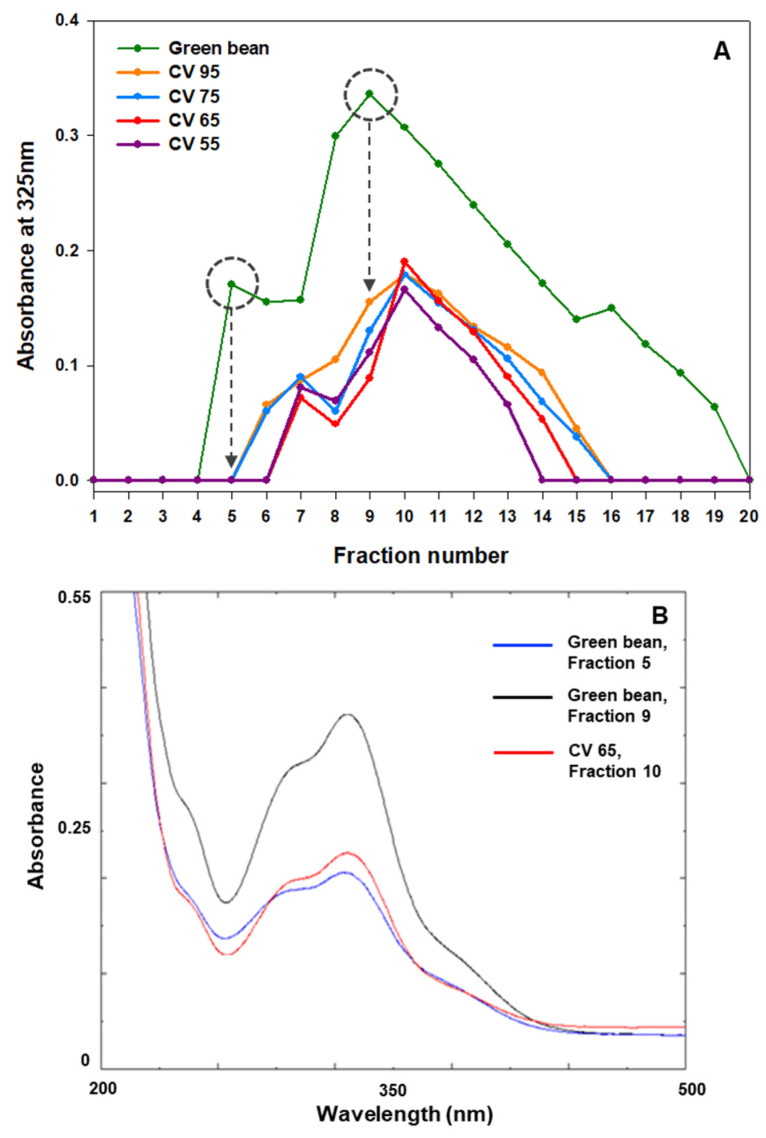
(**A**) HPLC fraction graph showing the thermal stability of chlorogenic acid present in green and roasted coffee bean extracts plotted at 325 nm using a UV–Vis spectrophotometer; (**B**) UV–Vis absorption spectra of fractions 5 (blue) and 9 (black) of green coffee bean extract, and fraction 10 (red) of coffee bean extract with a chromaticity value of 55 recovered from HPLC. The HPLC fraction volume was 1.5 mL each. CV, chromaticity value.

The HPLC-purified CGA-containing fractions were used to investigate thermal stability. In the case of green coffee beans, a broad fraction graph containing CGA with two peaks, fractions 5 and 9, was obtained from HPLC (Figure 6A). The presence of CGA in the collected HPLC fraction was confirmed using a UV–Vis spectrophotometer (Figure 6B). Based on the retention time, the first CGA fraction, fraction 5, is estimated to be a different kind of CGA than the CGA of fractions 9 and 10. Indeed, caffeoylquinic acid isomers(3-O-caffeoylquinic acid, 4-O-caffeoylquinic acid, and 5-O-caffeoylquinic acid) were isolated near fractions 9 and 10. As shown in Figure 6A, the chlorogenic acid of fraction 5 was severely damaged even at chromaticity value 95 (Light roasting degree). On the other hand, the chlorogenic acid of fraction 10 exhibited significant thermal stability at chromaticity value 55 (City roasting degree). This result suggests that thermal stability differs among CGA isomers.

### 3.6. LC-MS Analysis of CGA in the Fifth Fraction Recovered from HPLC

**Figure 7:**
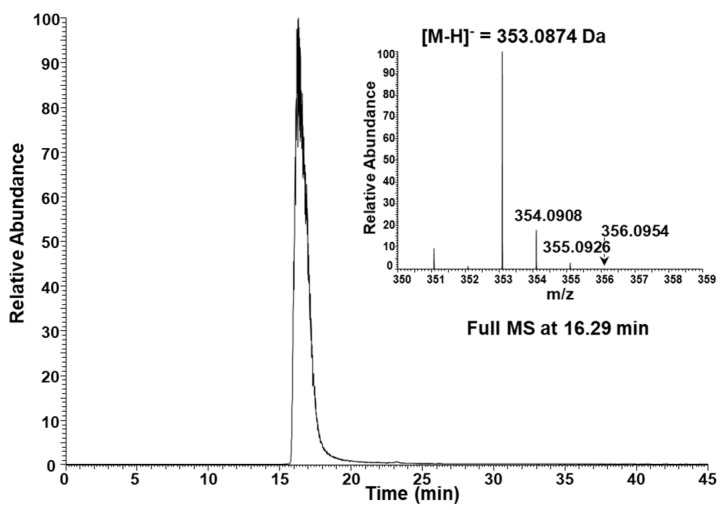
Extracted ion chromatogram and full MS spectrum of chlorogenic acid (theoretical exact mass: [M-H]^−^ = 353.0878 Da) in fraction 5.

The fifth HPLC fraction analyzed using a UV–Vis spectrophotometer was reanalyzed using LC-MS (Figure 7). Based on the MS spectrum, the CGA contained in fraction 5 was identified as one of the CGA isomers. In particular, the first of the four CGA peaks of Cameroon Blue Mountain green coffee bean extract identified by LC-MS (Figure 5B) matched exactly the chlorogenic acid in fraction 5. As described in Section 3.5, based on the retention time, the CGA contained in fraction 5 is estimated to be a different kind of CGA than the CGA of fractions 9 and 10.

## 4. Conclusions

This study confirmed the association between the acidity of the coffee bean extract and the roasting degree. The study findings revealed that the coffee bean extract pH decreased up to a chromaticity value of 75, beyond which it increased at higher chromaticity values. This phenomenon appears to be due to the degradation of CGAs and its decomposition product, caffeic acid. LC-MS analysis revealed the presence of four types of chlorogenic acids in the green coffee bean extract of Cameroon Blue Mountain. This study also revealed that thermal stability differs among CGA isomers.

## Data Availability

The original contributions presented in the study are included in the article. Further inquiries can be directed to the corresponding authors.
